# Supplementary material for: Spin-Hall nano-oscillator with oblique magnetization and Dzyaloshinskii-Moriya interaction as generator of skyrmions and nonreciprocal spin-waves
Source: Sci Rep. 2016 Oct 27;6:36020. doi: 10.1038/srep36020 (PMC5081538; doi:10.1038/srep36020)
Supplement: Supplementary Information [file srep36020-s9.pdf]

SUPPLEMENTARY INFORMATION

Spin-Hall nano-oscillator with oblique magnetization and Dzyaloshinskii-Moriya interaction as generator of skyrmions and nonreciprocal spin-waves

A. Giordano, R. Verba, R. Zivieri, A. Laudani, V. Puliafito, G. Gubbiotti, R. Tomasello, G. Siracusano, B. Azzerboni, M. Carpentieri, A. Slavin, and G. Finocchio

Supplementary note 1

Current density and Oersted field computation. The current density computation is formally based on the Ohm's law:

$$\mathbf{J} = \rho^{-1}\mathbf{E} \tag{S1}$$

where $\mathbf{E}$ is the local electric field and ρ is the resistivity of the material. The electric field is computed as the gradient field of the electrostatic potential V, so that:

$$\mathbf{E} = -\nabla V \tag{S2}$$

The charge conservation law yields $\nabla \cdot \mathbf{J} = 0$, so we get $\nabla \cdot (\rho^{-1}\nabla V) = 0$ with appropriate boundary conditions, that is, $\partial V / \partial \mathbf{n} = 0$ (where $\mathbf{n}$ is the normal to the boundary) except for the contacts, where there is a known current density entering or leaving the analyzed device (this entering/leaving current is one of the assigned data during the device simulation phase). This kind of elliptic problem in the electric scalar potential V is usually solved easily with Finite Element Method (FEM) for classical resistive device. However, for the examined device, further considerations are needed about the generation of an effective FEM mesh. Indeed, the thickness of few nano-meters of the two layers of CoFe and Pt, which have, on the other hand, a width and a length of some microns, makes the generation of a good quality mesh, a not immediate solvable problem, also taking into account the profile of the Au electrodes, which creates the electrical contact of the device. Efficient mesh generators could be used, but a direct meshing of the whole device usually leads to memory allocation problem and to the fault of the mesh generation, since

also irregular discretization requires a remarkable number of elements when the generated mesh must have a high quality. In other words, the tetrahedral elements are regular and small angles among the faces of the elements are possibly avoided in such a way to guarantee an accurate numerical solution of the problem. In order to overcome this kind of problems, we follow a strategy according to which the overall mesh is composed by small previously meshed blocks of various shapes: these blocks have a suitable correspondent surface discretization, allowing a direct fusion of them in the complete FEM mesh of the studied device. The mesh generator Tetgen [S1] has been adopted for the meshing of each block, since it allows us an easy management of the boundary discretization and allows the direct control of the mesh quality. The resulting so built mesh has more than 1.5 million of tetrahedral elements, which, according to the order (first or second) of the elements lead to a number of degree of freedom from about 5000000 to about 2500000, respectively. In Fig. S1 a mesh is plotted and a close up of the same mesh in the inter-electrodes regions show the different size of the elements in Au and in CoFe.

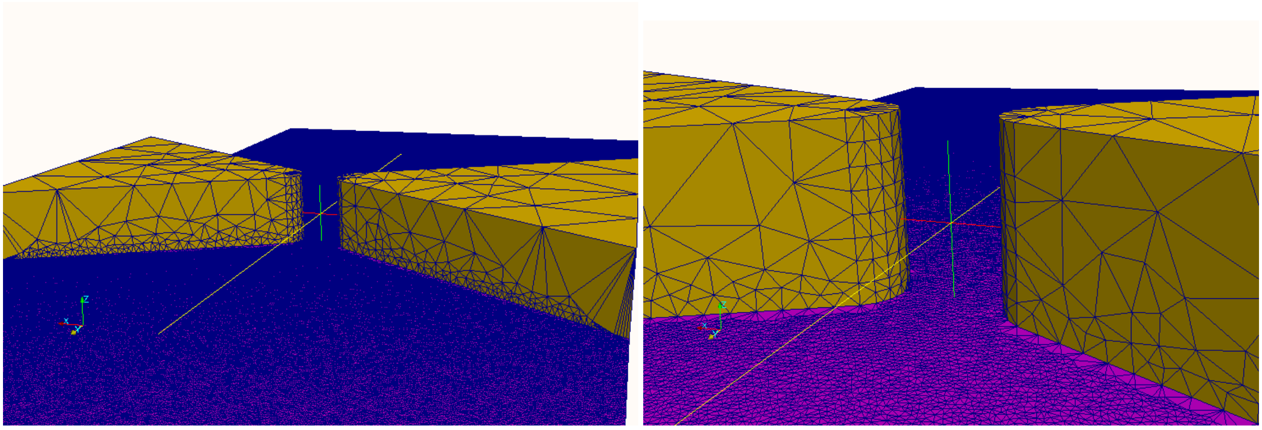

Figure S1 | FEM mesh visualization at the surface of the device and close up of the region between gold electrodes (yellow) and CoFe layer, showing clearly the difference in the size of the FEM elements due to the thickness of the CoFe and Pt layers.

As previously stated, the solution of this problem is expressed in terms of the electric scalar potential V and consequently a post-processing of the solution is necessary in order to estimate the current density distribution. In particular, the computation of the gradient of the electric scalar potential is required since $\mathbf{J} = \rho^{-1}\mathbf{E} = -\rho^{-1}\nabla V$ and this must be done for each cell of the finite element mesh. In order to avoid numerical errors this post-processing phase is performed accurately by exploiting the Regular Polyhedra Quadrature technique of the Poisson Integrals for the precise computation of the gradient of numerical solutions [S2]. From the current density thus computed in this way, it is also possible to compute the spatial distribution of the current by a numerical

integration $I = \int_S \mathbf{J} \cdot \mathbf{n} dS$, where S is a selected surface (for example in order to compute numerically the current flowing in CoFe layer, or in Pt layer, S must be selected as a surface cutting the device perpendicularly to x-axis at the centre of the device – see Fig. S1) and **n** is the unit vector perpendicular to S (clearly the sum of current flowing in the two layers is equal to the entering/leaving current in the Au electrodes). In addition, this current density distribution calculation is a necessary prerequisite for the calculation of Oersted field, that has been calculated as follows. Considering the computed current density distribution **J(**P**)** for every point P of the volume Ω, the Oersted field $\mathbf{H_c}$(Q) at point Q due to this current can be evaluated by direct integration over the volume discretized by the FEM mesh according to the expression:

$$\mathbf{H}_{\mathrm{c}}(\mathrm{Q}) = \nabla \times \frac{1}{4\pi} \int_{\Omega} \frac{\mathbf{J}(\mathrm{P})}{|\mathrm{P}-\mathrm{Q}|} d\Omega = -\int_{\Omega} \mathbf{J}(\mathrm{P}) \times \nabla G(\mathrm{P},\mathrm{Q}) d\Omega, \tag{S3}$$

where $\nabla\times$ is the curl operator, ∇ is the gradient operator and $G(\mathrm{P},\mathrm{Q}) = \frac{1}{4\pi|\mathrm{P}-\mathrm{Q}|}$ is the Green function for the considered problem. Although this kind of direct computation has a remarkable computational cost, it can be used with satisfying results and at the same time avoiding the implementation of the correspondent FEM problem for the Oersted field. Indeed, the latter suffers from several drawbacks linked to the boundary conditions assignment and can be equally prohibitive from the computational point of view due to the high number of degrees of freedom (in this last case higher due to the presence of vectorial unknowns).

Spin current density computation. The electrical current **J** flows through the Pt layer and, because of the spin-dependent scattering of the electrons, a spin current with a spin-polarization perpendicular to the direction of both charge current and spin current diffusion is created (spin-Hall effect). The spin-current direction is given by $\hat{z} \times \hat{\mathbf{J}}$, $\hat{\mathbf{J}}$ being the unit vector indicating the direction of the charge current. The spin current diffuses along the out-of-plane direction $\hat{z}$ exerting a Slonczewski like torque $\boldsymbol{\tau}_{\mathbf{SHE}}$ onto its magnetization:

$$\boldsymbol{\tau}_{\mathbf{SHE}} = -\frac{g\mu_B\alpha_H}{2\gamma_0 e M_S^2 t_{CoFe}} \mathbf{m} \times \mathbf{m} \times (\hat{z} \times \mathbf{J}), \tag{S4}$$

where g is the Landè factor, μ_B is the Bohr magneton, α_H is the spin-Hall angle, which represents the amount of charge current J converted into spin-current J_s, namely $J_s = \alpha_H J$. γ_0 is the

gyromagnetic ratio, e is the electron charge (e >0), M_S is the saturation magnetization of the ferromagnet, t_{CoFe} its thickness and $\mathbf{m}$ is the dimensionless magnetization of the ferromagnet.

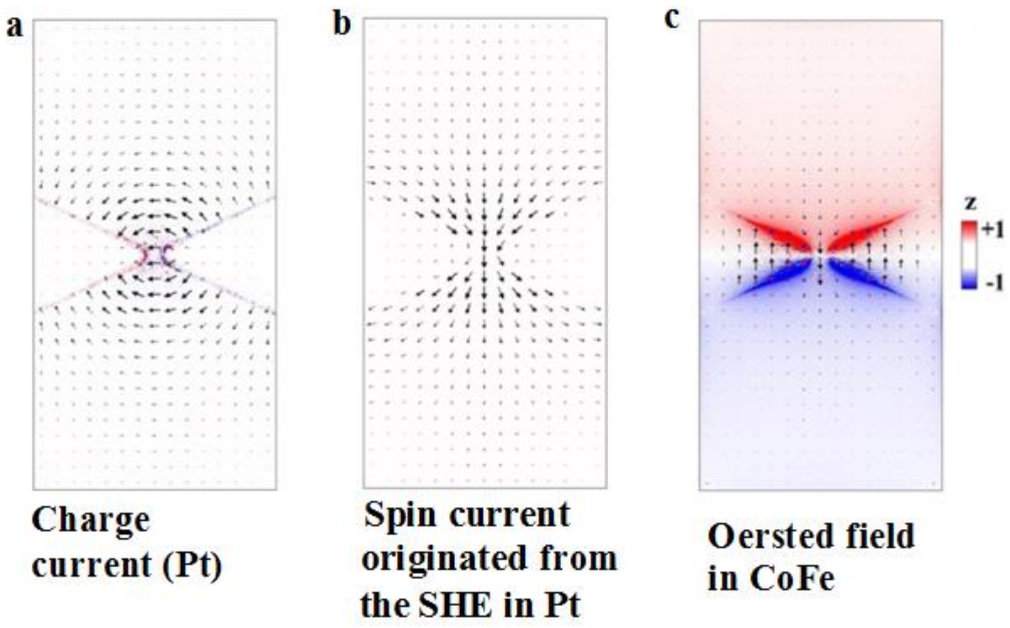

Figure S2 | Spatial distribution of charge and spin current, and Oersted field. a, charge current flowing in the Pt layer. **b,** spin current created by the spin-Hall effect in the Pt that gives rise to the spin-transfer torque applied to the CoFe layer. **c,** Oersted field in the CoFe layer generated by the whole charge current distribution. The arrow indicates the in-plane component of the vector while the color bar is related to its out-of-plane component (blue negative, red positive).

Supplementary note 2

Influence of *i*-DMI and Oersted field on the dynamics of the cylindrical mode. As discussed in the main text, the inclusion of *i*-DMI in the model induces a nonreciprocal effect in the propagation of cylindrical Slonczewski-like modes (SLM), mainly along the direction perpendicular to the in-plane projection of static magnetization, namely the *x* direction. This effect yields a difference between the wave vectors along the -*x* and +*x* directions, while the propagation remains symmetric along the *y* direction. In the absence of *i*-DMI, on the other hand, propagation is symmetric along both the *x* and the *y* direction. Fig. S3 shows the unique wave vector in this latter circumstance.

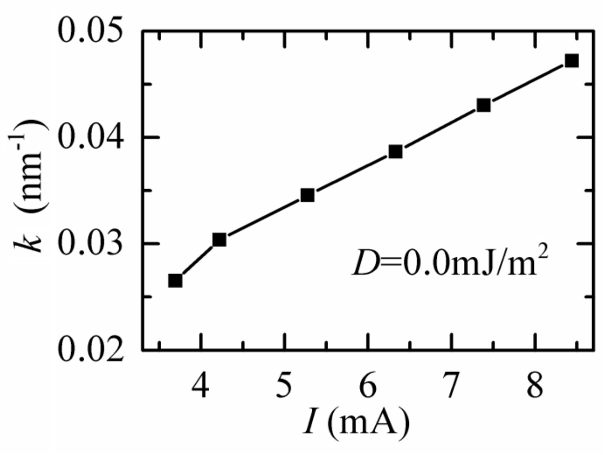

Figure S3 | Wave vector of cylindrical SLM. Wave vector as a function of the applied current in the absence of *i*-DMI corresponding to the wave vector along both the *x* and the *y* direction in the case of symmetric SLM.

Simulations performed for the phase diagram of Fig. 1c included the Oersted (Ampere) field. We also carried out simulation without this contribution to study its effect on the dynamics. Fig. S4, in particular, shows the frequencies of excited modes in the presence and absence of the Oersted field in the model, without *i*-DMI and with D=1.5mJ/m^2 and for two different values of distance between the Gold tips, d=100nm and 200nm, respectively. When *i*-DMI is not included in the model (Fig. S4a,c), the cylindrical modes are excited with a frequency that exhibits a blue-shift with the increase of the current. The influence of the Oersted field can be detected only for high values of the current, where frequencies obtained with Oersted field are slightly higher than the frequencies

obtained without including it in the model. When *i*-DMI is included in the model (Fig. S4b,d), on the other hand, it is possible to note a change in the trend of the frequency, which first increases with the increase of the current, then decreases for larger values of *I*. The Oersted field, also in this case, does not influence the output frequency for low values of current, whereas, for higher values it is possible to observe a non-monotonic behavior due to both the presence of the Oersted field and the excitation of strongly non-uniform modes, namely the spiral-like ones.

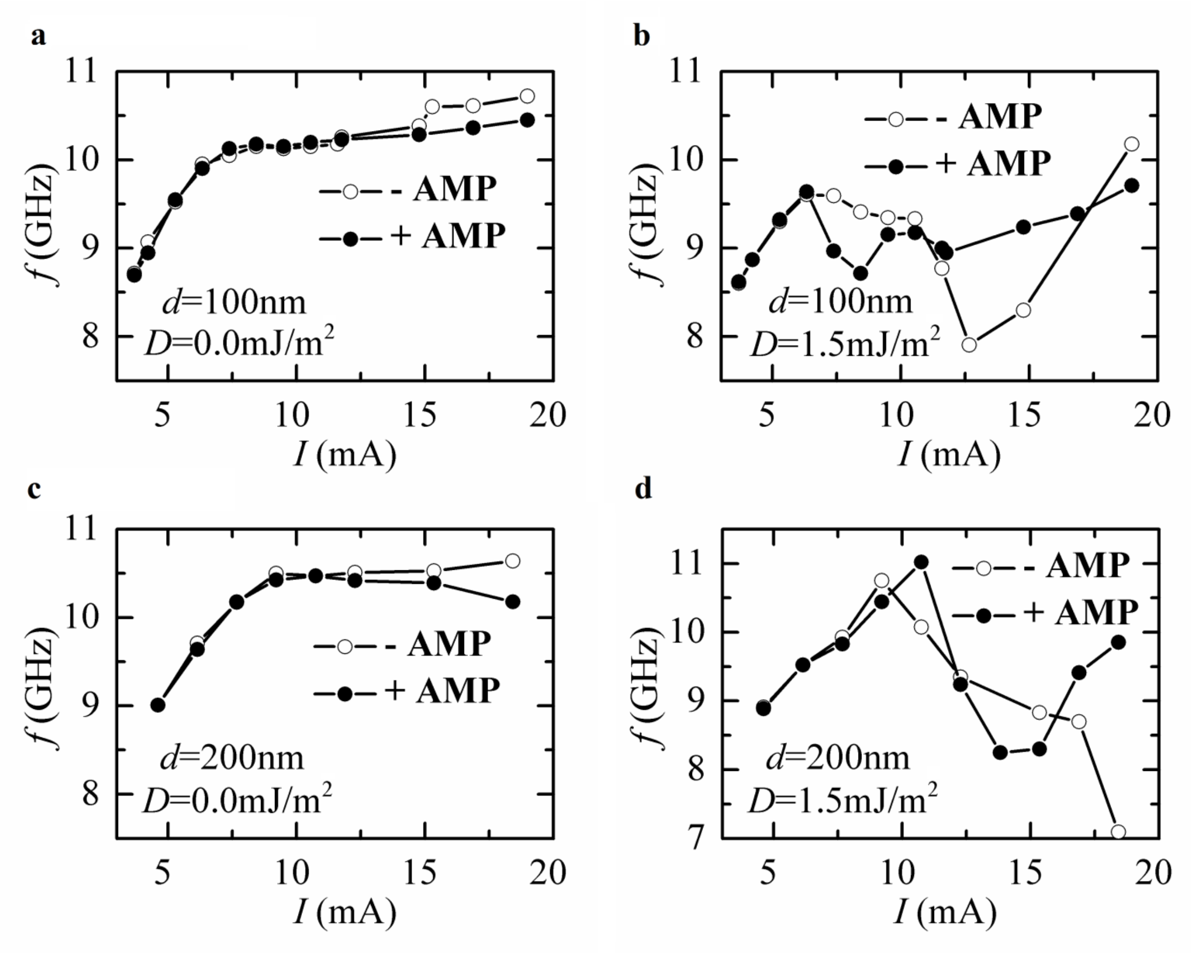

Figure S4 | Influence of *i*-DMI and Oersted field. Frequency vs. current for **a**, D=0.0mJ/m^2, d=100nm, **b**, D=1.5mJ/m^2, d=100nm, **c**, D=0.0mJ/m^2, d=200nm, **d**, D=1.5mJ/m^2, d=200nm. Each graph shows the obtained frequencies with and without the contribution of Oersted (Ampere) field indicated with "AMP".

[S1] Hang Si. 2015. TetGen, a Delaunay-Based Quality Tetrahedral Mesh Generator. ACM Trans. on Mathematical Software. **41** (2), Article 11 (February 2015), 36 pages. DOI=10.1145/2629697 http://doi.acm.org/10.1145/2629697.

[S2] Coco, S.; Laudani, A. Numerical differentiation of Laplacian 3-D FE solutions by using regular polyhedra quadrature of Poisson integrals. IEEE Trans. on Magnetics **37**, no.5, 3104-3107 (2001).

Legends for supplementary movies

Movie 1: Excited Slonczewski linear mode for I=4.22mA, B=400mT and D=0.0mJ/m^2. The colors indicate the y-component of the magnetization (blue negative, red positive).

Movie 2: Excited Slonczewski linear mode for I=5.28mA, B=400mT and D=0.0mJ/m^2. The colors indicate the y-component of the magnetization (blue negative, red positive).

Movie 3: Excited Slonczewski linear mode for I=4.22mA, B=400mT and D=1.5mJ/m^2. The colors indicate the y-component of the magnetization (blue negative, red positive).

Movie 4: Excited Slonczewski linear mode for I=5.28mA, B=400mT and D=1.5mJ/m^2. The colors indicate the y-component of the magnetization (blue negative, red positive).

Movie 5: Spiral mode for I=6.33mA, B=400mT and D=1.5mJ/m^2. The colors indicate the y-component of the magnetization (blue negative, red positive).

Movie 6: Single skyrmion nucleation process together with the excitation of propagating spin-waves for I=6.33mA, B=400mT and D=4.0mJ/m^2. The colors indicate the z-component of the magnetization (blue negative, red positive).

Movie 7: Spiral mode originated from a dynamical skyrmion exhibiting a rotational motion for I=6.33mA, B=400mT and D=1.5mJ/m^2. The colors indicate the z-component of the magnetization (blue negative, red positive).

Movie 8: Example of skyrmion gas for I=6.33mA, B=400mT and D=3.5mJ/m^2. The colors indicate the z-component of the magnetization (blue negative, red positive).
